# PTEN modulates EGFR late endocytic trafficking and degradation by dephosphorylating Rab7

**DOI:** 10.1038/ncomms10689

**Published:** 2016-02-12

**Authors:** Swapnil Rohidas Shinde, Subbareddy Maddika

**Affiliations:** 1 Laboratory of Cell Death and Cell Survival, Centre for DNA Fingerprinting and Diagnostics (CDFD), Nampally, Hyderabad 500001, India; 2 Graduate Studies, Manipal University, Manipal 576104, India

## Abstract

Tumour suppressor phosphatase and tensin homologue deleted on chromosome 10 (PTEN) is a lipid phosphatase that negatively regulates growth factor-induced survival signalling. Here, we demonstrate that PTEN attenuates epidermal growth factor receptor (EGFR) signalling by promoting late endosome maturation by virtue of its protein phosphatase activity. Loss of PTEN impairs the transition of ligand-bound EGFR from early to late endosomes. We unveil Rab7, a critical GTPase for endosome maturation, as a functional PTEN interacting partner. PTEN dephosphorylates Rab7 on two conserved residues S72 and Y183, which are necessary for GDP dissociation inhibitor (GDI)-dependent recruitment of Rab7 on to late endosomes and subsequent maturation. Thus, our findings reveal PTEN-dependent endosome maturation through phosphoregulation of Rab7 as an important route of controlling EGFR signalling.

## Abstract (web summary)

Rab7 is a critical GTPase for endosome maturation and it is implicated in the endocytic traffic of several receptors, including EGFR. In this study, the authors reveal the potential role of PTEN in the endocytic trafficking pathway of EGFR, which is dependent on its phosphatase activity through the direct post-translational modification of Rab7.

## Graphical abstract

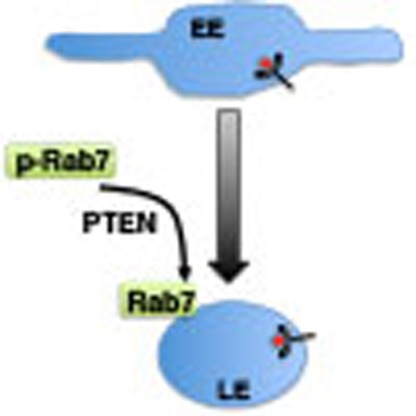

Phosphatase and tensin homologue deleted on chromosome 10 (PTEN) is an important tumour suppressor, which functions in many cellular processes such as cell proliferation, survival, growth, metabolism, migration and apoptosis^1^,^2^,^3^. PTEN was identified as a tumour-suppressor gene located at the chromosomal locus 10q23 and is found to be lost or mutated in various cancers such as glioblastomas, endometrial carcinomas, breast carcinomas and prostate carcinomas^2^,^4^. The manifestation of PTEN germline mutations leads to autosomal dominant syndromes such as Cowden syndrome, Bannayan Riley Ruvalcaba syndrome and Lhermitte Duclos diseases^5^. Functionally, PTEN is a dual-specific phosphatase that acts on both lipid and protein substrates^6^,^7^. The tumour suppressor function of PTEN is mostly attributed to its lipid phosphatase^2^,^7^,^8^,^9^. PTEN converts phosphatidylinositol-3,4,5-trisphosphate (PIP3) to phosphatidylinositol-4,5-bisphosphate (PIP2) at the cellular membrane and thereby negatively regulates oncogenic PI3K-AKT signalling^10^. However, the role of its protein phosphatase activity in controlling the oncogenic pathways was elusive.

EGFR (epidermal growth factor receptor) is a transmembrane receptor tyrosine kinase that modulates the rate of cell proliferation, growth and motility. Endocytic trafficking of growth factor receptor is one of the vital cellular mechanisms for spatial and temporal regulation of EGFR signalling^11^. Traditionally, on binding of ligand, the receptor–ligand complex is internalized via clathrin-dependent vesicles, which then delivers the complex to early endosomes for sorting^12^,^13^. The prevalent route of trafficking of the EGF/EGFR complex is into late endosomes by virtue of vesicle maturation, which is then followed by lysosomal fusion and degradation of the receptor^14^,^15^. Slowed kinetics of receptor degradation due to the defective endocytic pathway may account for EGFR overexpression in several cancers. Recently, PTEN was shown to be associated with cytoplasmic vesicles via phosphatidylinositol 3-phosphate (PI(3)P), an essential lipid of endosomes^16^. However, role of PTEN in endocytic trafficking pathway of growth factor receptors is unknown.

Here, we show that PTEN controls endocytic trafficking of EGFR by promoting late endosome maturation. PTEN is required for efficient transition of ligand-bound EGFR from early to late endosomes. Further we demonstrate that PTEN dephosphorylates Rab7 and regulates its localization to the late endosomal membranes, which is critical for endosome maturation.

## Results

### Depletion of PTEN delays EGFR trafficking to late endosomes

**Figure 1:**
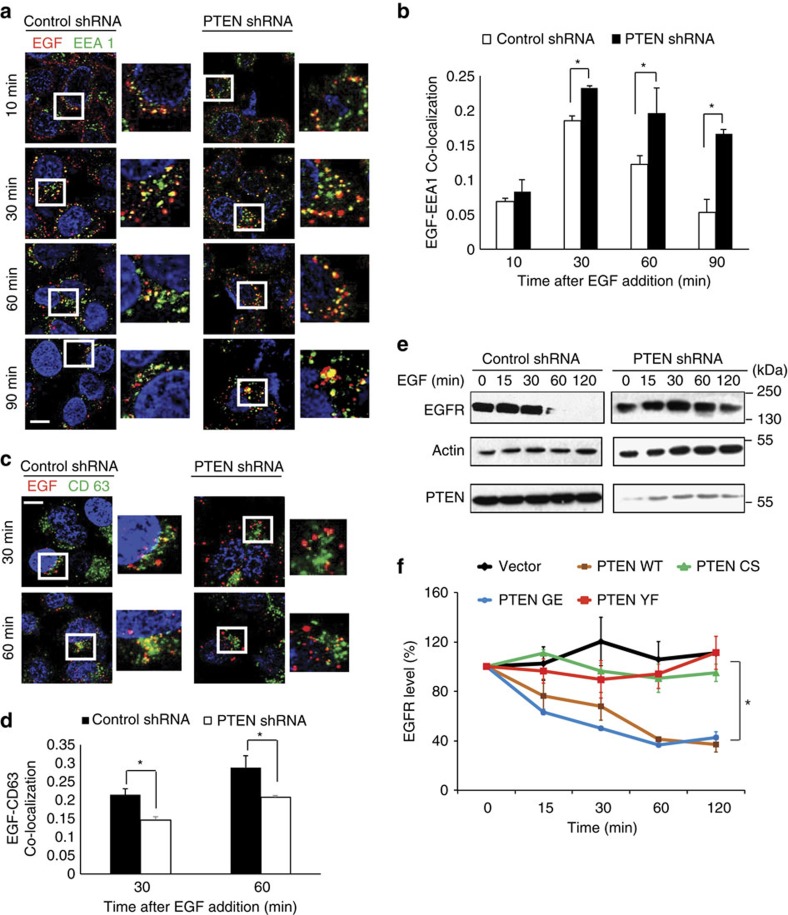
Loss of PTEN delays EGFR transport from early to late endosomes. (**a**) Serum-starved control or PTEN shRNA-transduced HeLa cells were pulsed with alexa Flour 647-conjugated EGF (100 ng ml^−1^) for 5 min. Cells fixed at different indicated times were imaged using confocal microscope after co-staining with antibodies against EEA1. Scale bar, 10 μm. (**b**) EGF-EEA1 co-localization in control and PTEN shRNA cells at different times after EGF addition was analysed by Manders method of pixel intensity correlation measurements using Image J/Fiji-Coloc2 plugin. Error bars indicate s.d. (*n*=100 cells for each time point from three independent experiments), *P<0.05 by Student's *t*-test. (**c**) The presence of EGF in late endosomes was analysed by co-staining with CD63 antibodies (scale bar, 10 μm) and (**d**) the extent of their co-localization in control and PTEN shRNA cells at 30 and 60 min post EGF addition was plotted (*n*=100 cells for each time point), Error bars indicate s.d. *P<0.05 by Student's *t*-test. (**e**) Overnight serum-starved control or PTEN shRNA transduced cells were treated with cycloheximide (50 μg ml^−1^) for 1 h and stimulated with 100 ng ml^−1^ EGF for indicated times. EGFR levels were tested by immunoblotting using specific antibody. (**f**) PTEN-deficient MDA-MB-468 breast cancer cells were transfected with PTEN WT (wild type), C124S (lipid and protein phosphatase dead), G129E (protein phosphatase active, lipid phosphatase dead) and Y138F (protein phosphatase dead, lipid phosphatase active) mutants. The EGFR levels at various times of post-EGF ligand addition is quantified using ImageJ and the levels of undegraded EGFR at each time point was calculated and plotted. Data plotted from three independent experiments; Error bars indicate s.d. *P<0.05, by Student's *t*-test.

To understand the role of PTEN in endocytic trafficking, we utilized alexa Flour 647-conjugated EGF to track the movement of ligand-bound receptors. We observed that depletion of PTEN resulted in significant accumulation of EGF signal over time compared with control cells (Supplementary Fig. 1A,B). To check if the accumulation of EGF in PTEN-depleted cells might be due to defective endocytic processing of ligand-bound receptor, we co-stained the cells with markers for different endocytic vesicles. Kinetics of EGF co-localization with early endosomal marker EEA1 in PTEN-depleted and control cells showed no changes in the initial time points suggesting an intact uptake and internalization of the receptor. However, localization of EGF with EEA1 positive endosomes was significantly increased in PTEN depleted HeLa cells at 60 and 90 min after internalization (Fig. 1a,b). On the other hand, staining with CD63, a late endocytic multivesicular body marker showed a significant decrease in co-localization of EGF with late endosomes in PTEN-depleted cells (Fig. 1c,d). These results showed that trafficking of EGF from early to late endosomes was hampered on PTEN depletion. As cargo movement from early to late endosomes is a critical step to promote the lysosome-dependent degradation of the internalized receptors, we next tested if the receptor degradation is prevented in PTEN-depleted conditions. In fact, loss of PTEN significantly delayed the ligand-induced degradation of EGF receptor compared with control cells (Fig. 1e). Also, a similar delay of ligand-induced degradation on PTEN loss was observed with fibroblast growth factor receptor (Supplementary Fig. 1C). Next, to determine whether the delay in EGFR degradation is dependent on PTEN catalytic activity we carried out EGFR degradation assay in PTEN-deficient MDA-MB 468 breast cancer cells. Expression of wild-type PTEN, but not the catalytically inactive C124S mutant, restored ligand-induced degradation of EGFR. Surprisingly, the protein phosphatase dead Y138F mutant but not lipid phosphatase dead G129E mutant failed to downregulate EGFR (Fig. 1f), suggesting that PTEN requires its protein phosphatase activity to promote maturation of EGFR-containing endosomes.

### Rab7 is a PTEN-associated protein

**Figure 2:**
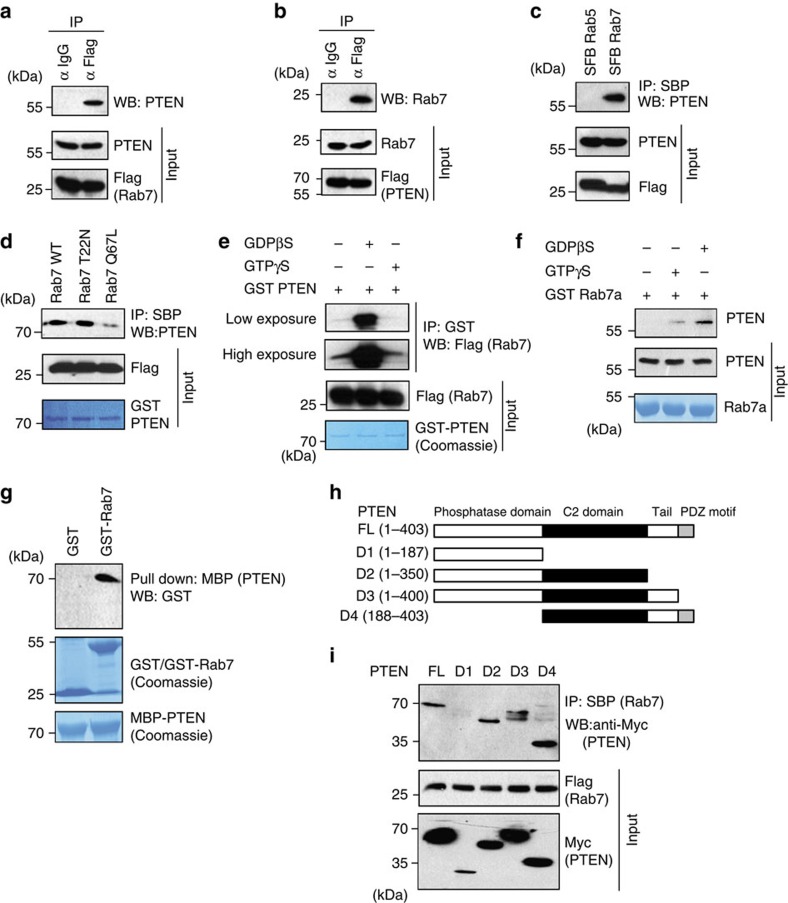
Rab7 is PTEN-associated protein. (**a**) HEK 293T cells transfected with the SFB-tagged Rab7 or (**b**) with SFB-tagged PTEN construct was subjected to immunoprecipitation (IP) with either control IgG or Flag antibody and the interaction of endogenous PTEN and Rab7 was determined by western blotting (WB) with their specific antibodies, respectively. (**c**) 293T cells were transfected with triple-tagged SFB-Rab5 or SFB-Rab7 and their interaction with PTEN was detected by immunoblotting with PTEN-specific antibody after immunoprecipitating with streptavidin (SBP) beads. (**d**) HEK293T cell lysates expressing SFB Rab7 WT or T22N (a dominant negative GDP bound) and Q67L (constitutively GTP-bound active) mutants were incubated with bacterially purified GST PTEN. The association of PTEN with Rab7 and its mutants was detected by immunoblotting with PTEN antibody. (**e**) SFB-Rab7 expressing HEK293T cell lysates pre-loaded either with GDPβS or GTPγS was incubated with glutathione–sepharose bound bacterially purified GST-PTEN and their binding was analysed by western blotting with Flag antibody. (**f**) GST Rab7 was loaded with either GTPγS or GDPβS *in vitro*. The association of PTEN with Rab7 was detected by immunoblotting with PTEN antibody after passing the 293T cell lysate through pre-loaded recombinant Rab7. (**g**) Agarose beads immobilized with bacterially expressed recombinant MBP-PTEN was incubated with either GST or GST-Rab7 proteins expressed in bacteria in the presence of GDP. The direct association of Rab7 with PTEN was detected by immunoblotting with GST antibody. Expression of the recombinant GST Rab7 and MBP-PTEN was shown by coomassie staining. (**h**) Schematic representation of N-terminal Myc-tagged PTEN (FL), along with its deletion mutants (D1–D4). (**i**) Myc-tagged PTEN constructs and SFB-Rab7 were co-expressed in HEK 293T cells, and the interaction of PTEN with Rab7 was detected by immunoblotting with anti-Myc antibodies after the cell lysates were pulled down with streptavidin beads.

As we noticed the importance of protein phosphatase activity of PTEN in EGFR trafficking, we next sought to identify its potential protein substrates in the cell. A catalytically inactive C124S version of PTEN was used as a substrate-trapping mutant. We established a HEK 293T derivative cell line stably expressing a triple-epitope (S-protein, Flag and streptavidin-binding peptide, SBP) tagged PTEN (SFB PTEN). Tandem affinity purification followed by mass spectrometry analysis enabled us to identify Rab7 as one among several PTEN-interacting proteins (Supplementary Table 1). Rab7, a member of the Ras superfamily of GTPases, plays an important role in the regulation of the trafficking, maturation, and fusion of endocytic and autophagic vesicles and has been shown to localize to the late endosomes and lysosomes^17^. Rab7 activity is essential for controlling aggregation and fusion of late endocytic structures and for maintenance of the perinuclear lysosome compartment^18^,^19^,^20^,^21^. Rab7 is critical for the transit of early endosomes to late endosomes and the transfer of cargo from late endosomes to lysosomes and thus regulates the lysosome-mediated degradation of activated EGF receptors^20^,^22^. We validated the interaction of PTEN-Rab7 *in vivo* through co-immunoprecipitation experiments. Endogenous PTEN was found to be associated with exogenously expressed SFB-tagged Rab7 (Fig. 2a) and conversely endogenous Rab7 was found to be immunoprecipitated with PTEN (Fig. 2b). PTEN interacts with Rab7 but not Rab5 confirming the specificity of their interaction (Fig. 2c). Since Rab7 cycles between cytosolic GDP-bound inactive and membrane-associated GTP-bound active conformations, we tested whether PTEN preferentially binds to particular guanine nucleotide-associated Rab7. PTEN exhibits a relatively stronger binding to a GDP-bound Rab7 T22N mutant protein and weaker affinity to GTP-bound Rab7 Q67L mutant protein (Fig. 2d). Further, *in vitro* binding assays in presence of non-hydrolyzable GTP (GTP-γ-S) or GDP (GDP-β-S) confirmed the preference of PTEN to associate with GDP-loaded Rab7 (Fig. 2e,f). Further, bacterially purified recombinant GST-Rab7 and MBP-PTEN proteins interacted with each other suggesting a direct interaction between PTEN and Rab7 (Fig. 2g). Analysis of binding region of PTEN by using various deletion mutants revealed that Rab7 interacts with the C2 domain of PTEN (Fig. 2h,i). Collectively, these data suggest that Rab7 is a PTEN-associated protein.

### PTEN dephosphorylates Rab7

**Figure 3:**
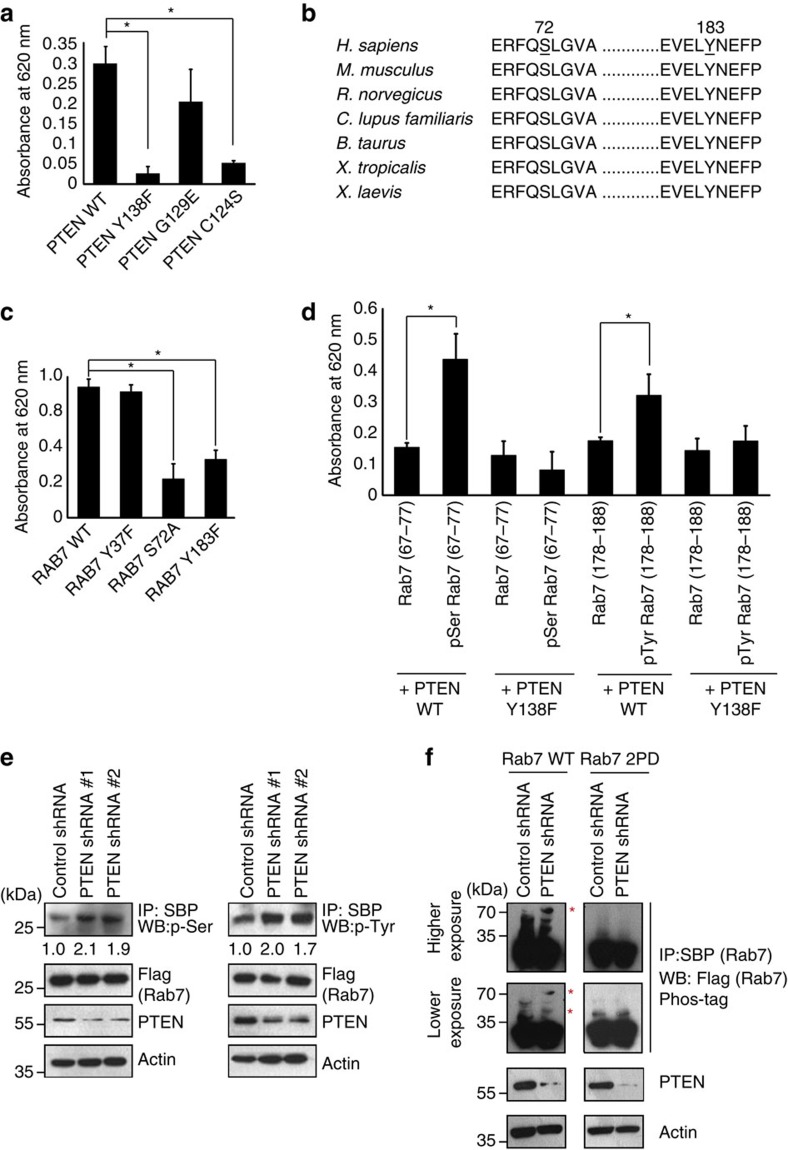
Rab7 is a substrate of PTEN protein phosphatase activity. (**a**) *In vitro* phosphorylated Rab7 was incubated with wild type and different catalytically inactive mutants of PTEN and the released phosphate was assayed colorimetrically using the malachite green reagent (absorbance values measured at 620 nm). Data represent mean absorbance from three independent experiments; *P<0.05, by Student's *t*-test. (**b**) Alignment of partial Rab7 protein sequences was shown. Conserved phosphorylation residues at serine 72 and tyrosine 183 (amino-acid positions in human protein indicated on top) shown bold and underlined. (**c**) Bacterially purified GST Rab7 WT and other indicated mutants were subjected to *in vitro* kinase assay using total 293T cell lysate. The phosphorylated Rab7 was then used as a substrate for PTEN phosphatase activity and the released phosphate was assayed colorimetrically using the malachite green reagent (absorbance values at 620 nm). Data represent mean absorbance from three independent experiments; *P<0.05, by Student's *t*-test. (**d**) The custom made Rab7 phosphopeptides (67–77 aa; QERFQpSLGVAF or 178–188 aa, TEVELpYNEFPE) along with control non-phosphorylated peptides were incubated with PTEN WT or PTEN Y138F mutant for 60 min at 37 °C. The released phosphate was assayed colorimetrically using the malachite green reagent (absorbance values at 620 nm); *n*=3, *P<0.05 by Student's *t*-test. (**e**) Control and two individual PTEN shRNA expressing cells transfected with SFB-Rab7 were subjected to immunoprecipitation with streptavidin (SBP) beads and immunoblotted with either phosphoserine (p-ser) or phosphotyrosine (p-tyr) antibodies. (**f**) Control and PTEN shRNA transduced HeLa cells were transfected with SFB Rab7 WT or Rab7 2PD mutant. Rab7 and its phosphorylated species (indicated with asterisk) were detected by using Phos-tag PAGE after immunoprecipitating Rab7 from the cells.

To test if Rab7 is a bonafide protein substrate of PTEN, we performed an *in vitro* phosphatase assay by using a phosphorylated Rab7. Wild-type PTEN, but not catalytically protein phosphatase dead C124S and Y138F mutants, dephosphorylated Rab7. A protein phosphatase proficient but lipid phosphatase dead G129E mutant could dephosphorylate Rab7 (Fig. 3a). Recent phosphoproteomic studies revealed several potential phosphorylation sites on Rab7 (refs 23, 24). Among these, two conserved S72 and Y183 sites (Fig. 3b) were highly enriched in the proteomic data sets. We found that mutation of S72 and Y183 sites significantly lowered the release of phosphate from Rab7 by PTEN (Fig. 3c). In fact, *in vitro* phosphatase assay using the custom peptides containing individual phosphorylation sites confirmed that PTEN dephosphorylates Rab7 at serine 72 and tyrosine 183 residues (Fig. 3d). Further evaluation of Rab7 phosphorylation in cells suggested that depletion of PTEN enhances the Rab7 phosphorylation at serine and tyrosine residues (Fig. 3e). Rab7 pull down followed by phosphate-affinity (Phos-tag) PAGE, where in phosphorylated Rab7 migrates slower, showed enhanced phosphorylation of Rab7 in PTEN-depleted cells (Fig. 3f). No phosphorylation was observed in Rab7 S72A/Y183F mutant, thus confirming these residues as target sites for PTEN. Together, this data reveal Rab7 as a protein substrate of PTEN.

### PTEN is required for Rab7 endosomal membrane targeting

**Figure 4:**
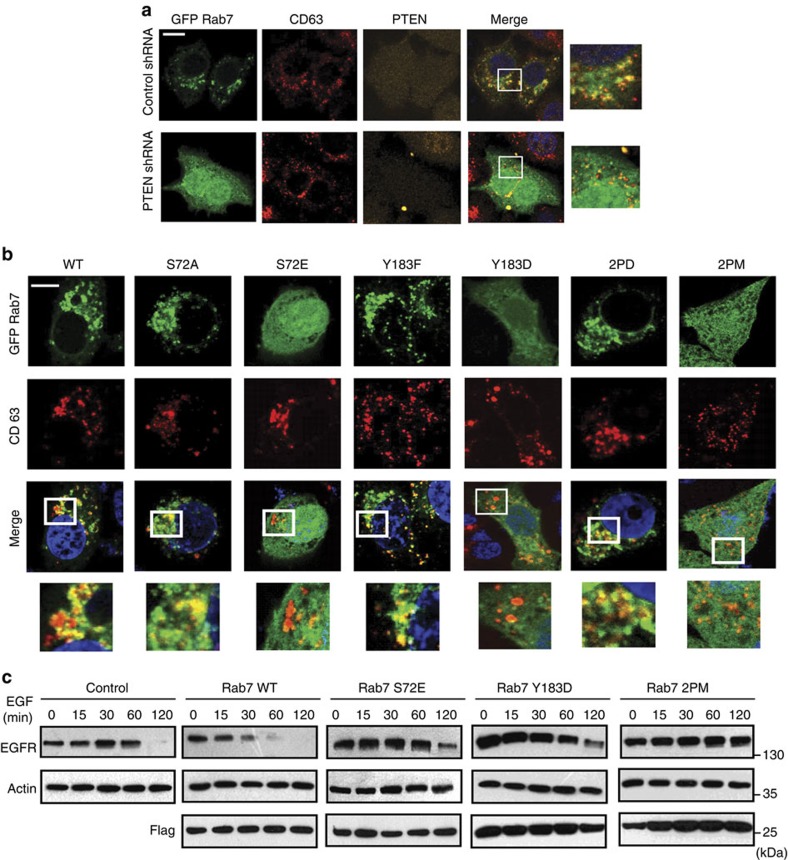
Dephosphorylation of Rab7 is required for its endosomal membrane localization. (**a**) HeLa cells expressing either control or PTEN shRNA were transfected with GFP-Rab7 and its localization to late endosomes/multivesicular bodies was determined by co-staining with CD63 antibody. Scale bar, 10 μm. (**b**) GFP-Rab7 wild type (WT), phospho-dead S72A, Y183F, S72A/Y183F (2PD) and phosphomimetic S72E, Y183D, S72E/Y183D (2PM) Rab7 mutants were transfected into HeLa cells and their endosomal localization was determined by confocal imaging after co-staining with CD63 marker. Scale bar, 10 μm. (**c**) 293T cells expressing either Rab7 WT, S72E, Y183D or Rab7 2PM mutant along with untransfected control cells were serum starved overnight. Cells were treated with cycloheximide (50 μg ml^−1^) for 1 h and stimulated with 100 ng ml^−1^ EGF for indicated times. EGFR levels were tested by immunoblotting using specific antibody.

Active Rab7 predominantly localizes to late endosomes and lysosomes^21^. To evaluate whether PTEN activates Rab7 by targeting to endosomal membranes, we carried out shRNA-mediated knockdown of PTEN in HeLa cells and checked its effect on Rab7 subcellular localization. Rab7 exhibits a distinct punctate vesicular staining in control cells but showed a diffused cytoplasmic localization in PTEN-depleted cells (Supplementary Fig. 2A,B). In fact, Rab7 fails to co-localize with CD63, a late endosomal marker, in PTEN-depleted cells (Fig. 4a). Wild-type Rab7 and a phospho-dead mutant (S72A/Y183F) could readily localize to late endosomes, whereas a constitutively phospho-mimetic mutant (S72E/Y183D) was precluded from endosomes (Fig. 4b). Interestingly, a single phosphomimetic substitution either at S72 or Y183 was sufficient to prevent Rab7 localization, thus suggesting that both residues need to be dephosphorylated by PTEN for efficient targeting of Rab7 on to endosomes. Co-staining with late endo-lysosomal marker LAMP2 further confirmed the defective endosomal membrane localization of Rab7 in PTEN-depleted cells (Supplementary Fig. 2C). Similarly, wild type and phospho-dead Rab7 mutants, but not phospho-mimetic mutants, effectively co-localized with LAMP2 (Supplementary Fig. 2D). As Rab7 phosphorylation perturbed its membrane targeting, we next tested if it effects EGFR trafficking. Expression of wild-type Rab7 accelerated EGFR degradation whereas phosphomimetic mutant significantly delayed EGFR turnover (Fig. 4c; Supplementary Fig. 3A,B). Expression of single phosphomimetic substitution either at S72 or Y183 was sufficient to delay EGFR turnover again suggesting the importance of PTEN-mediated dephosphorylation at both the residues. Since EGFR degradation is delayed by inefficient Rab7 membrane targeting, we next tested if it has any significance on the EGFR-mediated survival signalling pathway. In fact, expression of a phosphomimetic Rab7 mutant resulted in enhanced Akt activation (Supplementary Fig. 4A) followed by increased cell proliferation (Supplementary Fig. 4B), thus indicating that proper membrane targeting of Rab7 is important for optimal control of EGFR-induced cell survival signalling. Altogether, these results indicate that PTEN-mediated dephosphorylation of Rab7 is necessary for its appropriate membrane localization and function in EGFR trafficking through late endosomes.

### Dephosphorylated Rab7 interacts with its regulatory proteins

**Figure 5:**
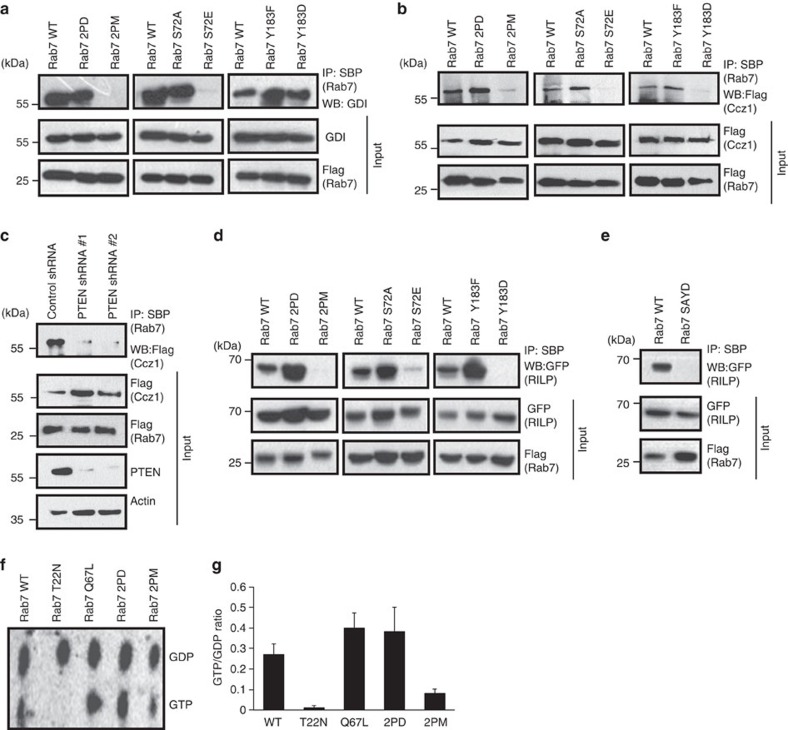
PTEN-mediated Rab7 dephosphorylation is necessary for its interaction with GDI, GEF and effector proteins. (**a**) HEK 293T cells transfected with either SFB-Rab7 WT or its various mutants were subjected to immunoprecipitation with streptavidin beads (SBP). The interaction of GDI with Rab7 and its mutants was analysed by immunoblotting with GDI-specific antibody. (**b**) Cells were transfected with SFB-Rab7 WT and its mutants along with Flag-tagged Ccz1 and the interaction of Rab7 with Ccz1 was determined by immunoblotting with Flag antibody after immunoprecipitating with SBP. (**c**) 293T cells expressing either control or two different PTEN shRNAs were co-transfected with SFB-Rab7 and Flag-Ccz1. The interaction of Ccz1 with Rab7 was analysed by immunoblotting with Flag antibody after immunoprecipitation with SBP beads. (**d**) Cells were transfected with SFB-Rab7 WT and its mutants along with GFP-tagged RILP and the interaction of Rab7 with RILP was determined by immunoblotting with GFP antibody after immunoprecipitating with SBP. (**e**) Cells were transfected with SFB-Rab7 WT and Rab7 S72A/Y183D mutant along with GFP-tagged RILP and the interaction of Rab7 with RILP was determined by immunoblotting with GFP antibody after immunoprecipitating with SBP. (**f**) Cells transfected with various indicated SFB-tagged Rab7 constructs were labelled with ^32^P-orthophosphate. GDP and GTP levels were analysed by thin layer chromotagraphy after immunoprecipitating Rab7 from the cell lysates by using streptravidin sepharose. (**g**) The GTP/GDP-bound ratio of various Rab7 mutants quantified by using Phosphorimager was plotted.

**Figure 6:**
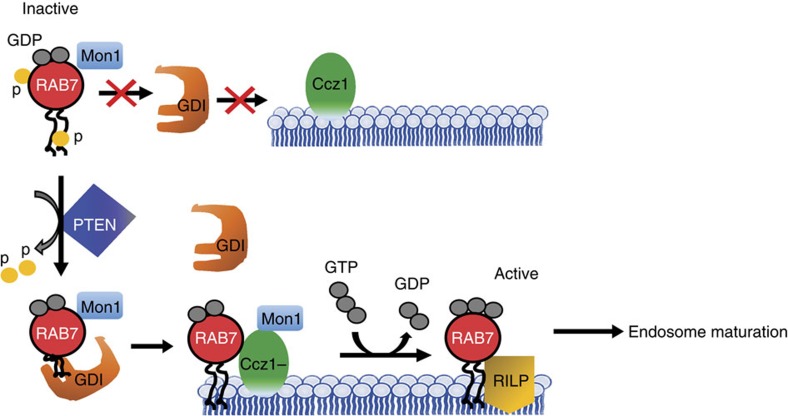
A model for PTEN-Rab7 association in endosome maturation. A proposed model to depict the role of PTEN in dephosphorylating inactive Rab7 in the cytosol, which facilitates its interaction with GDI for further presentation to Mon1-Ccz1 GEF activity at the endosomal membrane leading to active Rab7.

Membrane targeting of Rabs is a complex process orchestrated by several Rab-binding proteins. The existing model for Rab7 recruitment to endosomal membrane suggest that GDI (GDP dissociation inhibitor) complexes with cytoplasmic GDP-associated inactive Rab7 and delivers it to membrane where GDI displacement factor (GDF) displaces GDI and presents it to GEF (GDP/GTP exchange factor) for activation^25^,^26^,^27^,^28^. Once the activated Rab7 is stabilized on its cognate membrane its steady-state localization is dictated by binding to its downstream effector protein. To understand the mechanism of PTEN-mediated dephosphorylation on Rab7 membrane targeting, we first tested the ability of GDI interaction with Rab7. Wild type and phospho-dead (S72A/Y183F), but not phosphomimetic mutant (S72E/Y183D), interacts with GDI (Fig. 5a). Analysis with Rab7 single mutants suggests that ser^72^, but not tyr^183^ dephosphosphorylation, is critical for its interaction with GDI (Fig. 5a). On the other hand, dephosphorylation of both ser^72^ and tyr^183^ sites is necessary for its interaction with Ccz1, a component of GEF on the endosomal membrane (Fig. 5b). Also, depletion of PTEN results in loss of Rab7 binding to Ccz1 (Fig. 5c). In contrast, either depletion of PTEN (Supplementary Fig. 5A) or defective Rab7 (de)phosphorylation (Supplementary Fig. 5B) has no effect on Rab7 interaction with Mon1, a co-component of GEF. These data suggest that Mon1 binds constitutively with Rab7 irrespective of its state and localization, but ser^72^ dephosphorylation dictates Rab7 delivery to endosomal membrane where it could interact with Ccz1 for activation. Rab7 Tyr^183^ dephosphosphorylation is required for its stable interaction with GEF on the membrane post endosomal delivery by GDI. Recently, it has been shown that the hyper variable C terminus of Rab7 (180–183 residues) binds to its effector Rab-interacting lysosomal protein (RILP), which is essential for its stable insertion at the membrane^29^. A phosphomimetic Y183D mutant failed to interact with RILP (Fig. 5d). In addition to Rab7-reduced binding to RILP on PTEN depletion (Supplementary Fig. 5C), membrane delivery defective mutants S72E and 2PM mutants were also defective in binding to RILP (Fig. 5d), again supporting that Rab7 delivery to the membrane is a prerequisite for its interaction with its downstream effector. A phosphomimetic mutation of Y183D in the background of S72A, which was capable of translocating to membrane, was unable to interact with RILP (Fig. 5e) and was defective in RILP directed distinct aggregation at the endo/lysosomes (Supplementary Fig. 5D), clearly suggesting the importance of Y183 dephosphosphorylation by PTEN in stabilizing Rab7 association at the endosome. Further analysis of Rab7 mutants for their association with GDP and GTP revealed that the phosphomimetic mutant is predominantly in GDP-bound state, whereas the phospho-dead mutant occurs in GTP-bound state (Fig. 5f,g), thus clearly suggesting that dephosphorylation might be prerequisite for Rab7 activation. In conclusion, we identified that PTEN dephosphorylates Rab7 at S72 and Y183 residues, which is critical for its association with its GDI and subsequent delivery to endosomal membranes for activation by Mon1a–CcZ1 GEF complex, which in turn is required for late endosome maturation (Fig. 6).

## Discussion

PTEN is a well-known tumour suppressor and an important phosphatase that acts both on lipid and protein substrates. The catalytic role of PTEN to dephosphorylate phosphotidylinositol lipids and its importance as a negative regulator of PI3K signalling is very well established. However, the biological functions of PTEN can also be explained based on potentially additional mechanisms, including its protein phosphatase activity and catalytic activity-independent processes. Several recent studies have highlighted the significance of these alternate mechanisms. For example, protein phosphatase activity of PTEN has been shown to be important for controlling cell invasion, cell migration^30^, epithelial–mesenchymal transition^31^, platelet-derived growth factor receptor inactivation^32^ as well as regulation of *N*-methyl-D-aspartate receptor function^33^. On the other hand, in support of its catalytic activity-independent functions, PTEN has been shown to inhibit glioma cell migration through its C2 domain alone in the absence of catalytic domain^34^. Also, PTEN via its phosphatase-independent function has been shown to control p53 protein levels and its transcriptional activity *in vivo*^35^.

In this study, we revealed an important role for PTEN as a modulator of late endocytic trafficking through its protein phosphatase activity. We clearly demonstrated that PTEN dephosphorylates Rab7 and regulates its localization to the late endosomal membranes. Rab7 is a central player in the endocytic pathway, where it governs the transition of early to late endosomes followed by their fusion with lysosomes^17^. Depletion of Rab7 in the cell leads to trapping of cargos in late endosomes and results in the accumulation of enlarged and densely packed late endosomes/multivesicular bodies^20^, thus underlying the importance of Rab7 in transferring cargo to lysosomes. Several mechanisms have been identified to regulate the activity of Rab7 in cells, mainly through interaction of various regulatory proteins. Normally inactive form of GDP-bound Rab7 resides in the cytosol bound to its GDI. Through the concerted action of GDI, GDF and GEF, Rab7 is activated on the endosomal membrane. But the knowledge about the role of post-translational modifications in regulating Rab7 function is very limited. So far to our understanding, C-terminal prenylation^36^, which is critical for its membrane insertion is the only documented Rab7 modification in the cell. Our study provides a regulatory mechanism of Rab7 through dephosphorylation at two conserved residues, which is critical for its recruitment on to the endosomal membranes. While we identified PTEN as a phosphatase for removal of the phosphate groups, it would be critical in future studies to identify the kinases that phosphorylate these residues and keep Rab7 in an inactive state until the receipt of an appropriate activation signal.

As a core regulator of PI3-kinase signalling network, PTEN suppresses EGFR-mediated cell growth and proliferation signalling. By activating Rab7-mediated endosome maturation, PTEN provides an alternate mechanism for spatial and temporal control of EGFR signalling in cells, the aberration of which may lead to pathological conditions. The existence of several tumour-derived PTEN Y138 mutants^7^,^30^, which selectively lacks protein phosphatase activity, emphasizes the importance of lipid phosphatase-independent cellular processes during PTEN-mediated tumour suppression. In tumours containing catalytically inactive PTEN, the control over Rab7-dependent EGFR endosomal degradation may be lost leading to uninterrupted growth signalling, and this could have important implications for tumour progression.

## Methods

### Plasmids

Full-length PTEN was cloned into Myc, GST, myelin basic protein (MBP) and SFB (S-protein/Flag/SBP)-tagged destination vectors using the Gateway cloning system (Invitrogen). The point mutants for PTEN were generated by PCR-based site-directed mutagenesis and cloned into MBP- and Myc-tagged destination vectors. Mammalian cDNAs for Rab5 and Rab7a was a kind gift from Dr Rashna Bhandari (Centre for DNA Fingerprinting and Diagnostics, Hyderabad, India). Rab5 and Rab7a were cloned to SFB-triple-tagged vector using Gateway cloning system. The point mutations for Rab7 were generated by PCR-based site-directed mutagenesis and cloned into SFB-, GST- and GFP-tagged destination vectors. mRFP Rab5 and EGFP Rab7a were kindly provided by Dr Marino Zerial (Max Planck Institute of Molecular Cell Biology and Genetics, Germany). Flag-tagged Mon1a and Ccz1 were kindly provided by Dr Jason M. Kinchen and Dr Kodi Ravichandran (University of Virginia). GFP RILP was a kind gift from Dr Mahak Sharma (IISER, Mohali, India).

### Antibodies and reagents

The following commercial antibodies were used in this study: anti-PTEN (1:1,000, WB) and anti-Rab7 (1:1,000, WB) (Cell Signalling Technology), anti-EEA1 (1:100, IF) (Abcam), anti-EGFR (1005, Sc-03) (1: 5,000, WB), anti-Myc (9E10) (1:1,000), anti-GFP (1:1,000, WB) (all from Santa Cruz Biotechnology), anti-Flag (1:10,000, WB and IF) and anti-actin (1:10,000, WB) (both from Sigma), anti-GDI (1:2,500, WB) (Invitrogen), anti-LAMP2 (1:100, IF) and anti-CD63 (1:100, IF) (both from DSHB), anti-phospho serine (1:500), anti-phospho tyrosine (1:1,000, WB) (Millipore). HRP-conjugated anti-Mouse and anti-Rabbit secondary antibodies were obtained from Jackson Immunological. Alexa flour-647-conjugated EGF (Invitrogen), unlabelled EGF, GDPβS, GTPγS (all from Sigma) were used.

### Cell lines and transfection

HEK 293T, HeLa, BOSC23 and MDA-MB-468 cell lines were used in this work. All cell lines were purchased from American Type Culture Collection, which were tested and authenticated by the cell bank using their standard short tandem repeats (STR)-based techniques. Cells were also continuously tested for mycoplasma contamination by using 4',6-diamidino-2-phenylindole (DAPI) staining. HEK 293T or HeLa were transfected with various plasmids using PEI (Polysciences) according to the manufacturer's protocol. Briefly, the plasmid diluted in serum-free RPMI medium was mixed with PEI (1 μg μl^−1^) in 1:3 ratio. After incubating the DNA–PEI mixture at room temperature (RT) for 15 min, the complexes were added to cells to allow the transfection of plasmid.

### Tandem affinity purification

PTEN-associated proteins were isolated by using tandem affinity purification as described before^37^,^38^. Briefly, 293T cells stably expressing SFB–PTEN C124S substrate trapping mutant were lysed with NETN buffer (20 mM Tris-HCl at pH 8.0, 100 mM NaCl, 1 mM EDTA, 0.5% Nonidet P-40) containing 1 μg ml^−1^ of each of pepstatin A and aprotinin on ice for 20 min. The cell lysates were added on to streptavidin–sepharose beads (Amersham Biosciences) and incubated for 1 h at 4 °C. After removing the unbound proteins by washing the beads thrice with lysis buffer, the associated proteins were eluted using 2 mg ml^−1^ biotin (Sigma) for 60 min at 4 °C. The elutes from the first step of purification were then incubated with S-protein–agarose beads (Novagen) for 1 h at 4 °C. After clearing the unbound proteins by washing, the proteins associated with S-protein–agarose beads were eluted by boiling in SDS-loading buffer for 10 min at 95 °C. The proteins were identified by mass spectrometry analysis carried out by the Taplin biological mass spectrometry facility at Harvard University.

### Immunoprecipitation

For immunoprecipitation assays, cells were lysed with NETN buffer as described above. The whole-cell lysates obtained by centrifugation were incubated with 2 μg of specified antibody bound to either protein A or protein G–sepharose beads or with streptavidin–sepharose beads (Amersham Biosciences) for 1 h at 4 °C. The immunocomplexes were then washed with NETN buffer four times and applied to SDS–PAGE.

### Western blotting

Western blotting was carried out by following standard protocols. Protein (30 μg) was separated by denaturing SDS–PAGE and then transferred on to polyvinylidene difluoride membranes. The membranes were blocked in 5% non-fat dried milk in Tris-buffered saline (TBS) and then incubated overnight with the primary antibodies at 4 °C. Then, the blots were incubated with the corresponding secondary antibodies conjugated with HRP at RT for 1 h. Visualization was carried out by enhanced chemiluminescence detection (Thermo Fisher Scientific). Uncropped raw version of various blots is included in Supplementary Fig. 6.

### RNA interference and lentiviral infection

Control siRNA and prevalidated PTEN siRNA were purchased from Qiagen (catalogue no. SI00301504). Transfection was carried out twice 30 h apart with 200 nM siRNA using Oligofectamine reagent according to the manufacturer's protocol (Invitrogen). PTEN shRNA (clones purchased from Open Biosystems) were transfected transiently using PEI (Invitrogen) in BOSC23 packaging cells along with packaging vectors. Forty-eight hours post transfection, the viral medium was collected and added to the target cells along with polybrene (8 μg ml^−1^). Forty-eight hours post infection, cells were collected and processed for various assays and immunoblotting was performed with the specific antibodies to check the efficiency of knockdown.

### Immunofluorescence staining

Cells grown on coverslips were fixed with 3% paraformaldehyde solution in PBS containing 50 mM sucrose at RT for 15 min. After permeabilization with 0.5% Triton X-100 buffer containing 20 mM HEPES at pH 7.4, 50 mM NaCl, 3 mM MgCl_2_ and 300 mM sucrose at RT for 5 min, cells were incubated with a 1% BSA for blocking at RT for 60 min. After washing with PBS, cells were incubated with primary antibodies for 2–3 h at RT followed by three times washes with 1 × PBS 10 min each. Then, cells were incubated with FITC or Rhodamine-conjugated secondary antibodies at RT for 60 min followed by three times wash with 1 × PBS 10 min each. Nuclei were counterstained with DAPI. After a final wash with PBS, coverslips were mounted with glycerine containing paraphenylenediamine. Images were taken using confocal microscope (LSM Meta 510, Zeiss).

### *In vitro* phosphatase assay

Bacterially expressed GST Rab7 was incubated with 1 mM ATP and HEK293T cell lysate and reaction buffer (25 mM HEPES, pH 7.5, 25 mM β-glyserophosphate, 25 mM MgCl_2_, 2 mM dithiothreitol (DTT) and 0.1 mM sodium orthovanadate) at 37 °C for 90 min. Following the incubation, beads were washed two times with NETN. The phosphorylated Rab7 was incubated with PTEN wild type or PTEN catalytically inactive mutants in reaction buffer (25 mM HEPES, pH 7.4, 1 mM EDTA and 10 mM DTT) at 30 °C for 60 min. The released phosphate was detected using Malachite Green Assay Kit (Cayman) by measuring the absorbance at 620 nm.

### EGF uptake assay and immunofluorescence and quantification of EGF assays

PTEN shRNA-transduced HeLa cells were grown on coverslips and subjected to serum starvation overnight. To follow EGF internalization, the cells were precooled on ice for 10 min and serum-free medium containing 100 ng ml^−1^ EGF-647 was added. EGF-647 was bound to cells for 1 h at 4 °C and they were shifted to 37 °C for 5 min. After 5 min of internalization, the medium was changed to complete medium (RPMI with 10% donor bovine serum (DBS)), and the cells were incubated at 37 °C for up to 2 h. Subsequently, the internalization was stopped by shifting the cells back to 4 °C and surface-bound EGF was removed with high salt and low pH buffer (0.2 M sodium acetate and 0.5 M NaCl at pH 4.5). Further, the cells were fixed with 3% paraformaldehyde for 15 min at RT. After permeabilization with 0.2% triton X-100, the cells were stained with necessary antibodies. Further the coverslips were mounted and the imaging was done using confocal microscope (LSM 700, Zeiss). Z-stacks with 0.5-μm step size were acquired over a total imaging distance of 15 μm. To quantify EGF-647 signal and co-localization with EEA1 and CD 63, Manders method of pixel intensity correlation measurements was performed using the Image J/Fiji-Coloc2 plugin Fiji software.

### Phos-tag assay

Phos-tag acrylamide (Wako Chemicals) based gel analysis was carried out to detect phosphorylated species of Rab7. PTEN-depleted HeLa cells were transfected with SFB-Rab7 wild type or SFB-Rab7 2PD mutants. Twenty-four hours post transfection, cells were lysed and subjected to immunoprecipitation using streptavidin–sepharose beads. The protein samples eluted by boiling were run on 12% polyacrylamide gel containing 50 μM Phos-tag acrylamide (Wako Chemicals) and 50 μM MnCl_2_. After electrophoresis, Phos-tag acrylamide gels were washed with gentle shaking in transfer buffer containing 1 mM EDTA for 15 min and then incubated in transfer buffer containing 0.01% SDS without EDTA for 15 min according to the manufacturer's protocol. Proteins were then transferred to polyvinylidene difluoride membrane for further analysis by standard immunoblotting protocol using specific antibodies.

### EGFR degradation assay

HeLa cells were maintained in serum-free RPMI medium overnight. Next day, the serum-starved cells were treated with 50 μg ml^−1^ cycloheximide for 1 h to prevent synthesis of new EGFR during stimulation by EGF. Cells stimulated with 100 ng ml^−1^ EGF for different indicated times were collected and lysed using NETN lysis buffer. The lysates were subjected to SDS–PAGE and levels of undegraded EGFR were determined by western blotting. Using ImageJ software, the intensity of EGF or EGFR at each time point was calculated relative to the intensity measured at 15 min (which was set to 100%). Similar degradation assay was also performed with FGF1 (100 ng ml^−1^) along with 20 U ml^−1^ heparin.

### *In vitro* binding assays

GST Rab7 was loaded with either GTPγS (1 mM) or GDPβS (1 mM) in binding buffer (150 mM NaCl, 5 mM EDTA and 25 mM Tris-HCl, pH 7.4) at 37 °C for 30 min. Then preloaded GST Rab7 was incubated with HEK 293T cell lysates containing 10 mM MgCl_2_, for 2–4 h at 4 °C. Further, the beads were washed with wash buffer (150 mM NaCl, 2.5 mM MgCl_2_ and 25 mM Tris-HCl, pH 7.4) and subjected to western blot analysis with respective antibodies. For direct binding assays, bacterially expressed GST Rab7 or GST protein cell lysates were incubated with bacterially purified MBP-PTEN bound to Dextrin sepharose (GE Healthcare) for 2 h at 4 °C. The bound complexes were washed and eluted by boiling in SDS sample buffer. Western blot analysis was performed to detect the complexes using specific antibodies.

On the other hand, HEK 293T cell lysates containing 20 mM EDTA expressing SFB Rab7 were incubated with either GTPγS (1 mM) or GDPβS (1 mM) for 30 min at RT. Then the tubes were placed on ice and loading was stopped by adding 10 mM MgCl_2_. Further, the loaded lysates were incubated with GST PTEN at 4 °C for 2–4 h and the beads were washed with wash buffer and subjected to western blot analysis with respective antibodies.

### Determination of the GTP: GDP ratio of Rab7

The analysis was performed essentially as described earlier^39^. Briefly, HEK 293T cells were transfected with Rab7 WT, Rab7 T22N, Rab7 Q67L, Rab7 2PD or Rab7 2 PM mutants, in phosphate-free Dulbecco's modified Eagles medium containing 0.5 mCi per ml [32P]orthophosphate. Sixteen hours post transfection, the cells were washed three times with cold PBS and then lysed with NETN buffer (20 mM Tris-HCl at pH 8.0, 100 mM NaCl, 1 mM EDTA, 0.5% Nonidet P-40) containing 1 μg ml^−1^ of each of pepstatin A and aprotinin on ice for 20 min. The cell debris were removed by centrifugation and crude cell lysates were incubated with streptavidin–sepharose beads (Amersham Biosciences) for 1 h at 4 °C. The beads were washed five times in NETN lysis buffer and the bound nucleotides were then eluted in 6 μl sample buffer (2 mM EDTA, 2 mM DTT, 0.2% SDS, 5 mM GDP and 5 mM GTP) for 2 min at 70 °C. Four μl of the samples were spotted onto 0.1 mm PEI-cellulose TLC plates (Merck), which were developed for 40 min in 0.6 M Na-phosphate, pH 3.4. The plates were dried and placed in autoradiography cassettes containing Phosphoimager sheet. A Phosphorimager (Applied Biosystems) was used to determine the GTP: GDP ratios, taking into account that the specific activity of [^32^P] GDP is 2/3 that of [^32^P] GTP.

## Additional information

**How to cite this article:** Shinde, S. R. and Maddika, S. PTEN modulates EGFR late endocytic trafficking and degradation by dephosphorylating Rab7. *Nat. Commun.* 7:10689 doi: 10.1038/ncomms10689 (2016).

## Supplementary Material

Supplementary Information Supplementary Figures 1-6 and Supplementary Table 1
