# Supplementary material for: PTEN modulates EGFR late endocytic trafficking and degradation by dephosphorylating Rab7
Source: Nat Commun. 2016 Feb 12;7:10689. doi: 10.1038/ncomms10689 (PMC4754336; doi:10.1038/ncomms10689)
Supplement: Supplementary Information — Supplementary Figures 1-6 and Supplementary Table 1 [file ncomms10689-s1.pdf]

Supplementary information

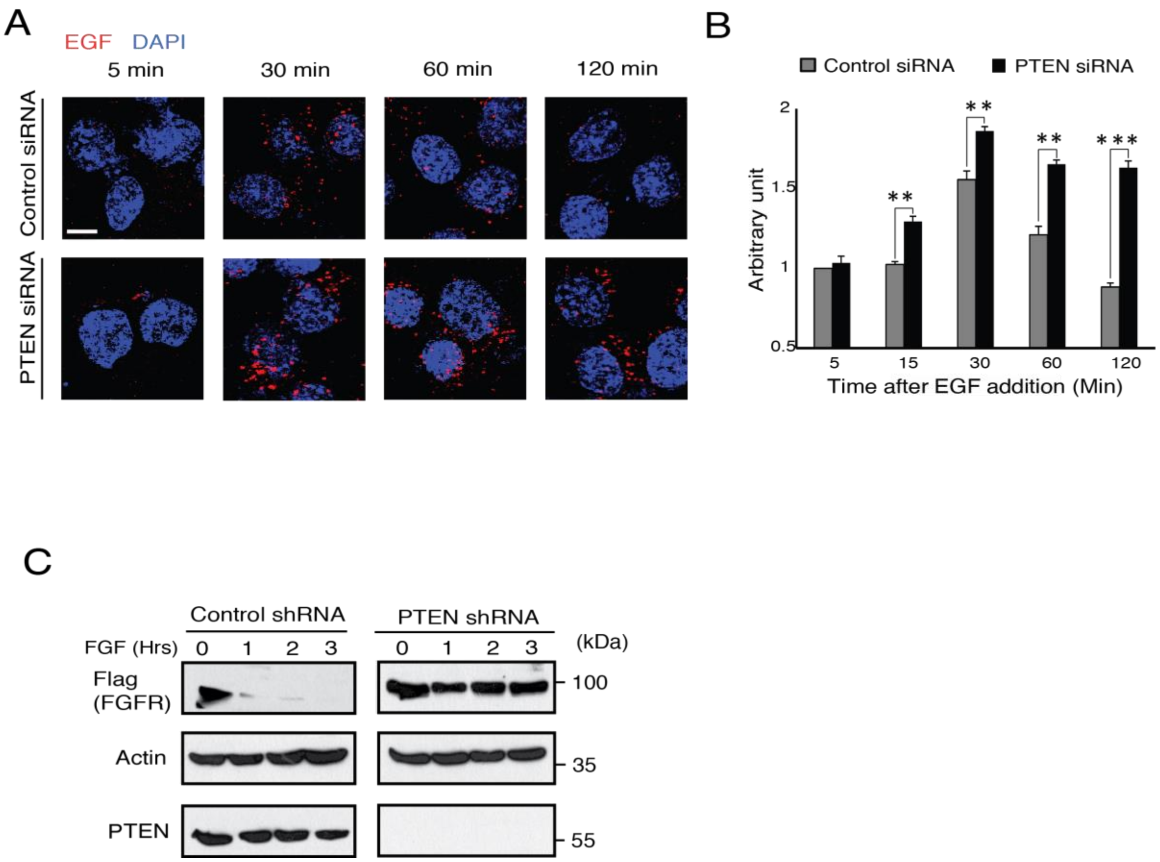

Supplementary Figure 1: PTEN promotes efficient degradation of growth factor receptors. (A) Overnight serum starved control or PTEN siRNA-transfected HeLa cells were pulsed with alexa Flour 647-conjugated EGF (100 ng/mL) for 5 minutes. Cells were fixed at different indicated times and the levels of internalized EGF were analysed by using scanning confocal microscope. Scale bar, 10µm. **(B)** The mean A647-EGF signal per cell was quantified and normalized to control siRNA at 5 minutes of EGF addition. Data is represented from three independent experiments; **P < 0.01, ***P < 0.001, students t-test. **(C)** Cells expressing Flag-tagged FGFR transduced with either control or PTEN shRNA were treated with cycloheximide (50µg/ml) for 1 hour and stimulated with 100 ng/mL FGF for indicated times. FGFR levels were tested by immunoblotting using specific antibody.

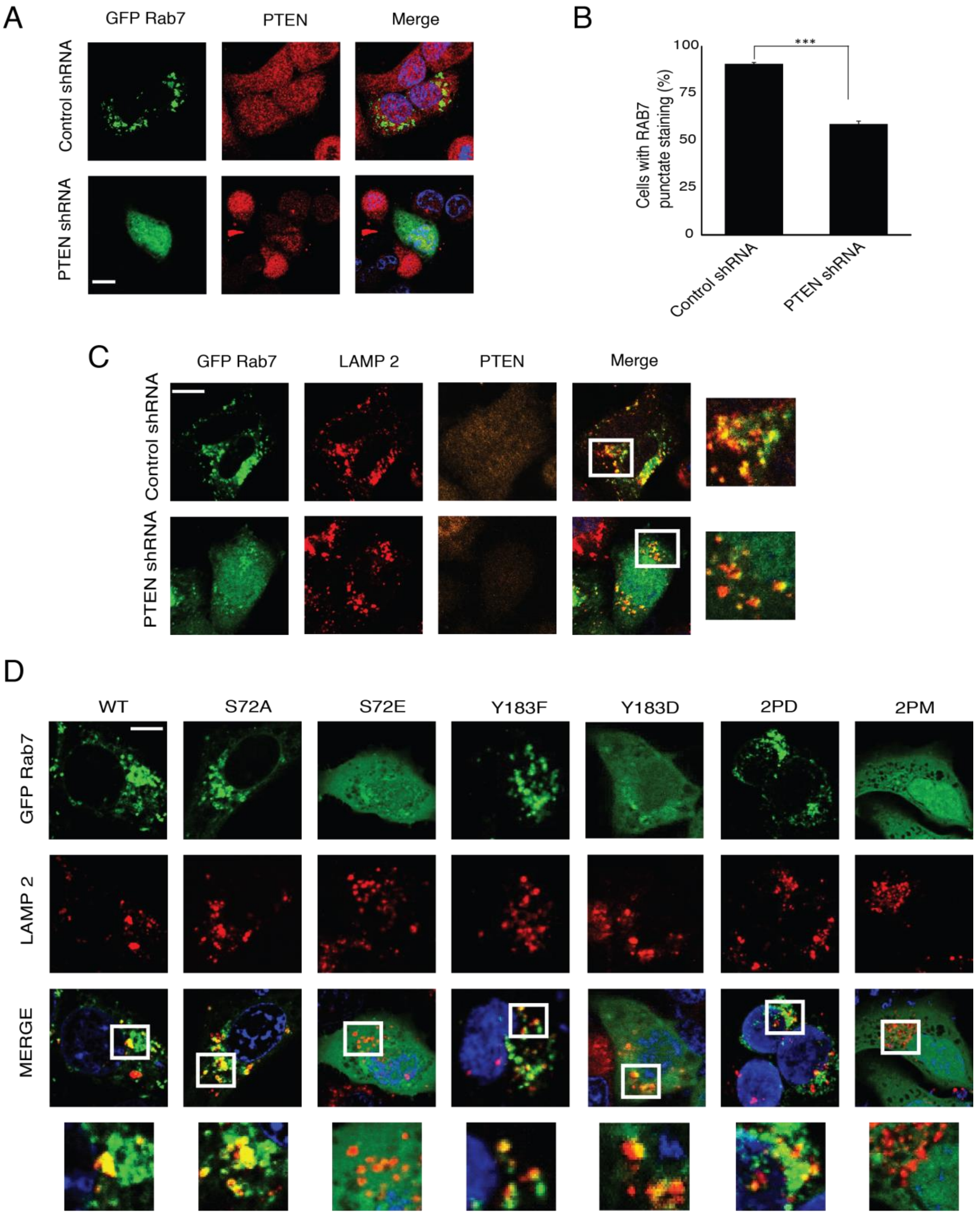

Supplementary Figure 2: Dephosphorylation of Rab7 is necessary for its endosomal membrane localization (A) HeLa cells transduced with either control or PTEN shRNA were transfected with GFP-Rab7. Rab7 localization to punctate vesicular structures was analyzed by

immunoflouresce using confocal microscopy. Scale bar, 10μm. **(B)** The number of cells with punctate Rab7 staining were counted in PTEN positive and negative cells and the data is represented from 100 cells for each condition; *P < 0.001 by student's t-test. **(C)** HeLa cells expressing either control or PTEN shRNA were transfected with GFP-Rab7 and its localization to late endosomes/lysosomes was determined by co-staining with LAMP2 antibody. Scale bar, 10μm. **(D)** GFP-Rab7 wild type (WT), phospho-dead S72A, Y183F, S72A/Y183F (2PD) and phosphomimetic S72E, Y183D, S72E/Y183D (2PM) Rab7 mutants were transfected into HeLa cells and their late endolysosomal localization was determined by confocal imaging after co-staining with LAMP2 marker. Scale bar, 10μm.

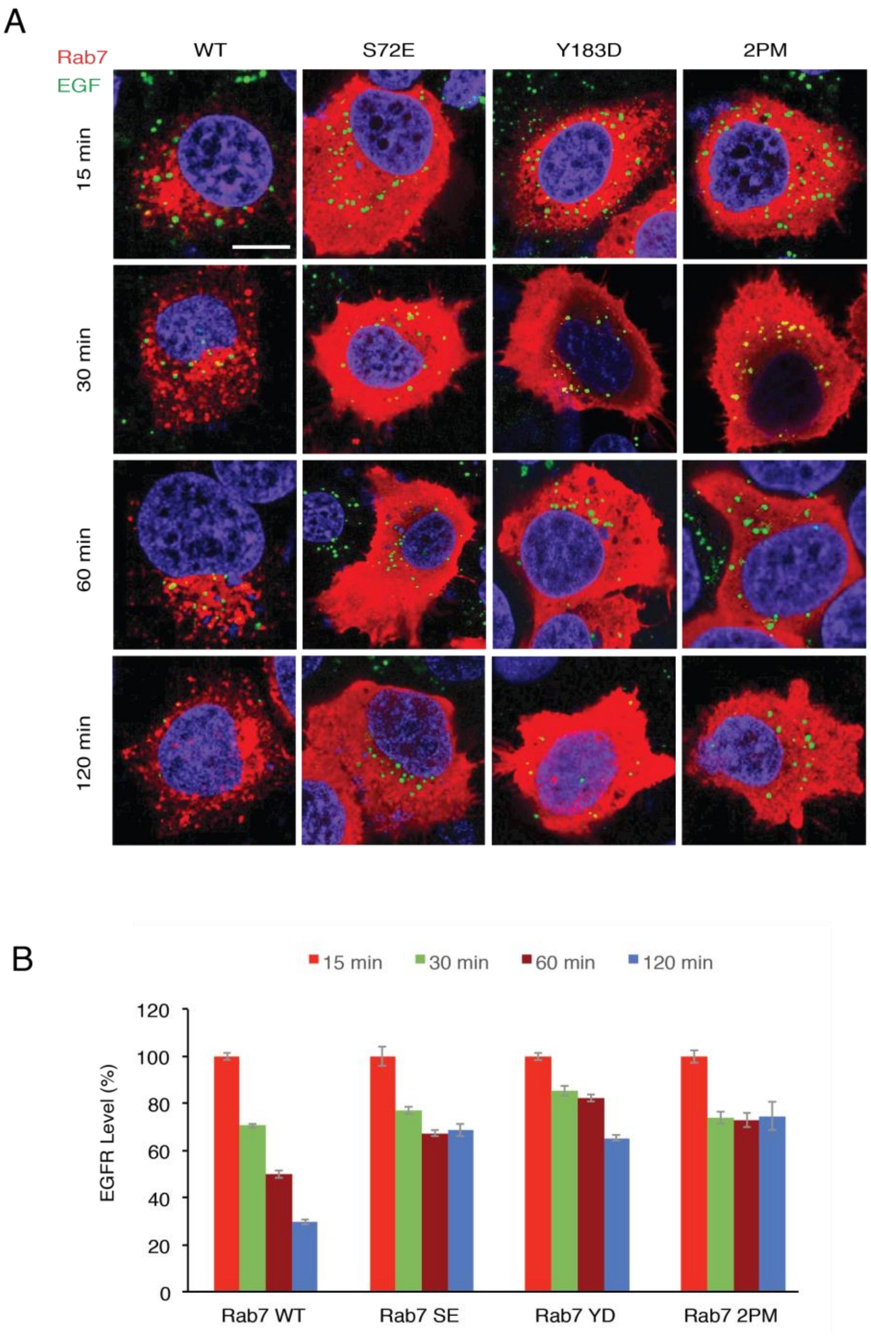

Supplementary Figure 3: Rab7 dephosphorylation is necessary for efficient degradation of EGFR (A) Cells expressing wild type Rab7 and various mutants were pulsed with alexa Flour 647-conjugated EGF (100 ng/mL) for 5 minutes. Cells were fixed at different indicated times and the levels of internalized EGF were analysed by using scanning confocal microscope. Rab7 expression is indicated by Red staining and internalized EGF foci was shown in green by applying pseudocolour. **(B)** The mean A647-EGF signal per cell was quantified and normalized to control siRNA at 15 minutes of EGF addition. Data is represented from three independent experiments.

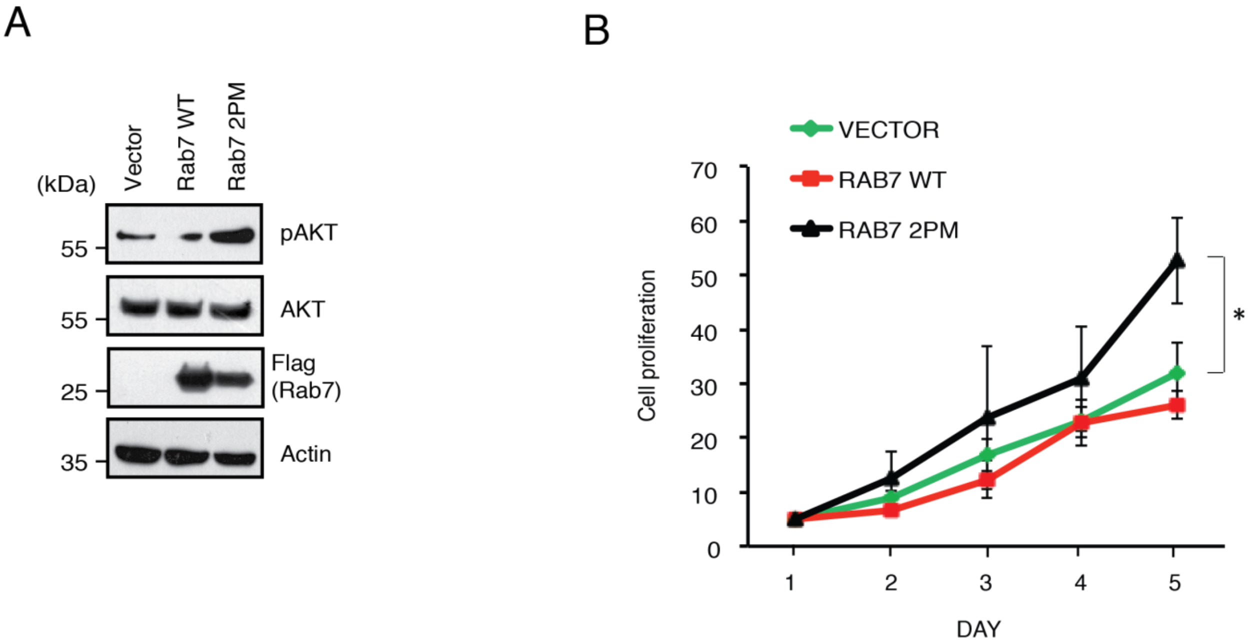

Supplementary Figure 4: Constitutively phosphorylated Rab7 promotes cell proliferation. (A) HeLa Cells were transfected with vector, wild type Rab7 or Rab7 2PM mutant, and the activation of Akt is determined by blotting with phospho-specific antibody along with other indicated antibodies. **(B)** Cells expressing wild type Rab7 or 2PM mutant were seeded and cell proliferation was measured by tryphan blue exclusion for 5 days. Error bar indicates standard deviation (n=3), P<0.01; students *t-test.*

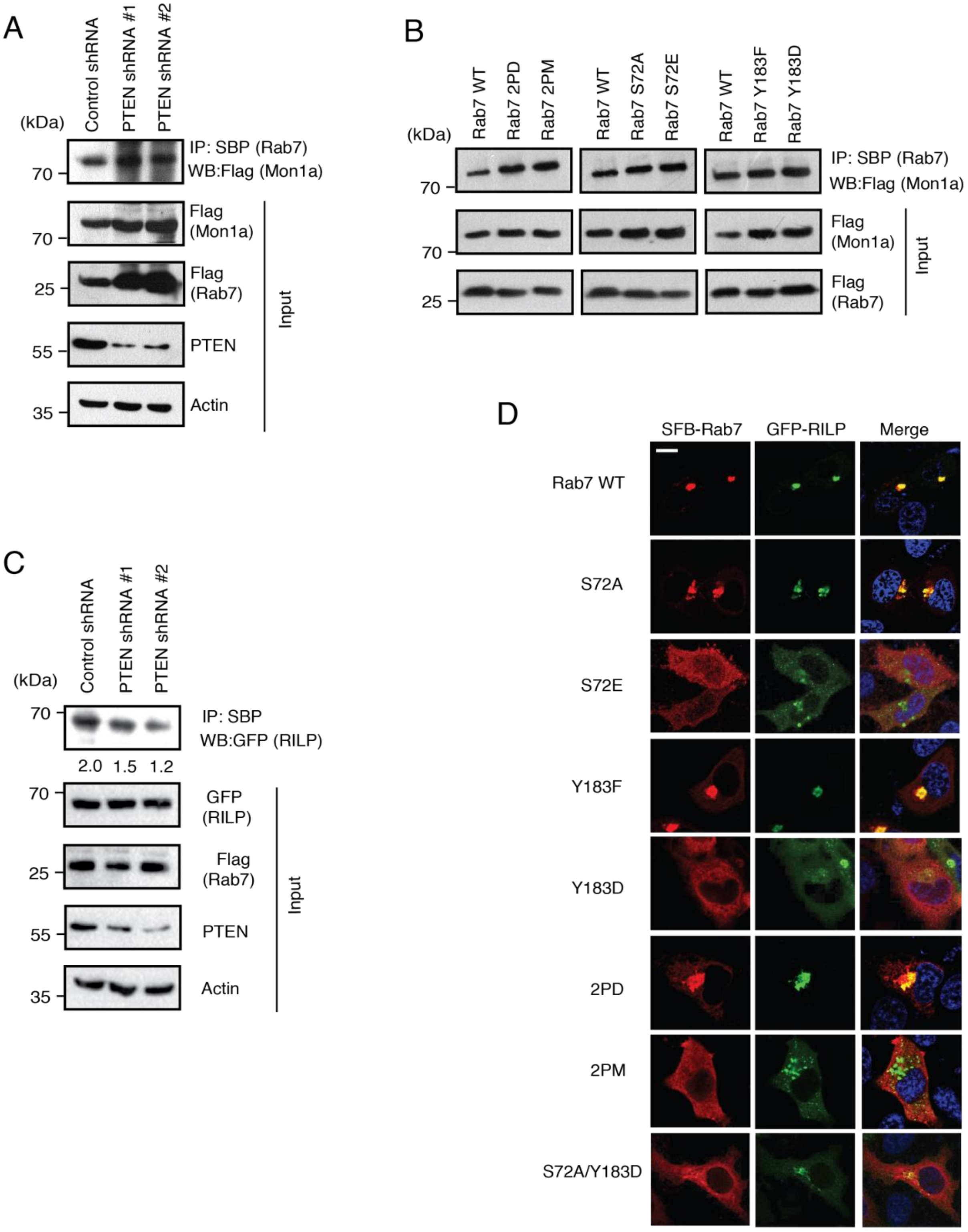

Supplementary Figure 5: PTEN mediated Rab7 dephosphorylation is necessary for its interaction with GDI, GEF and effector proteins. (A) 293T cells expressing either control or two different PTEN shRNAs were co-transfected with SFB-Rab7 and Flag-Mon1a. The

interaction of Mon1a with Rab7 was analyzed by immunoblotting with Flag antibody after immunoprecipitation with SBP beads. **(B)** Cells were transfected with SFB-Rab7 WT and its mutants along with Flag-tagged Mon1a and the interaction of Rab7 with Mon1a was determined by immunoblotting with Flag antibody after immunoprecipitating with streptavidin beads. **(C)** 293T cells expressing either control or two different PTEN shRNAs were co-transfected with SFB-Rab7 and GFP-RILP. The interaction of RILP with Rab7 was analyzed by immunoblotting with GFP antibody after immunoprecipitation with SBP beads. **(D)** Triple tagged SFB-Rab7 wild type (WT), phospho-dead S72A, Y183F, S72A/Y183F (2PD) and phosphomimetic S72E, Y183D, S72E/Y183D (2PM) Rab7 mutants were transfected into HeLa cells along with GFP-RILP and their endosomal co-localization was determined by confocal imaging after co-staining with Flag antibody.

Figure 1 blots

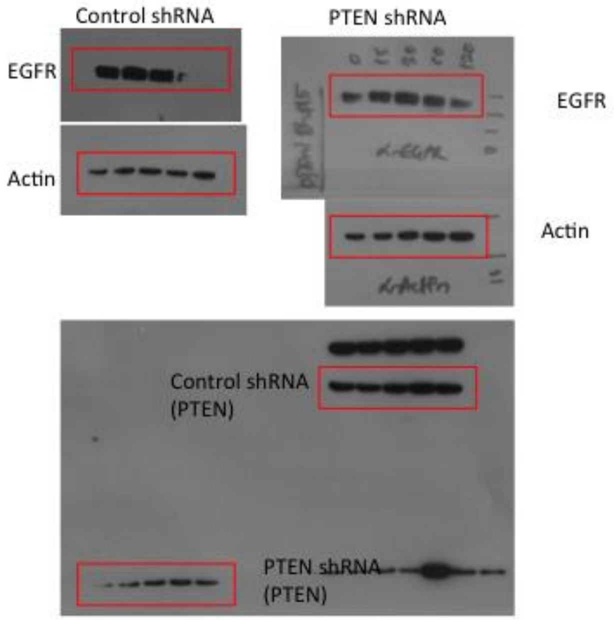

Figure 2 blots

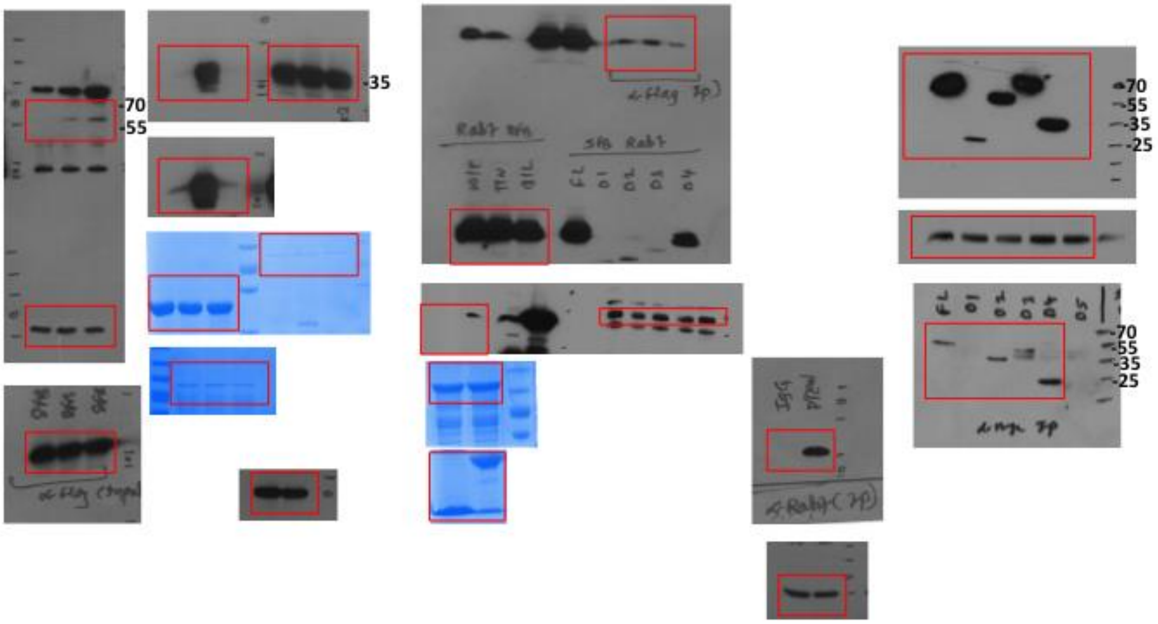

Figure 3 blots

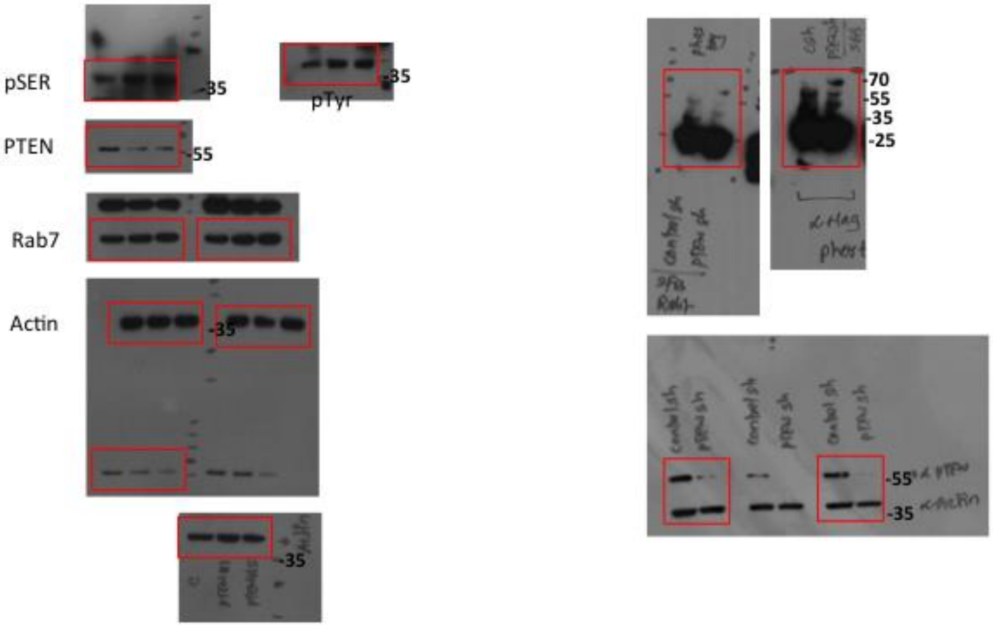

Figure 4 blots

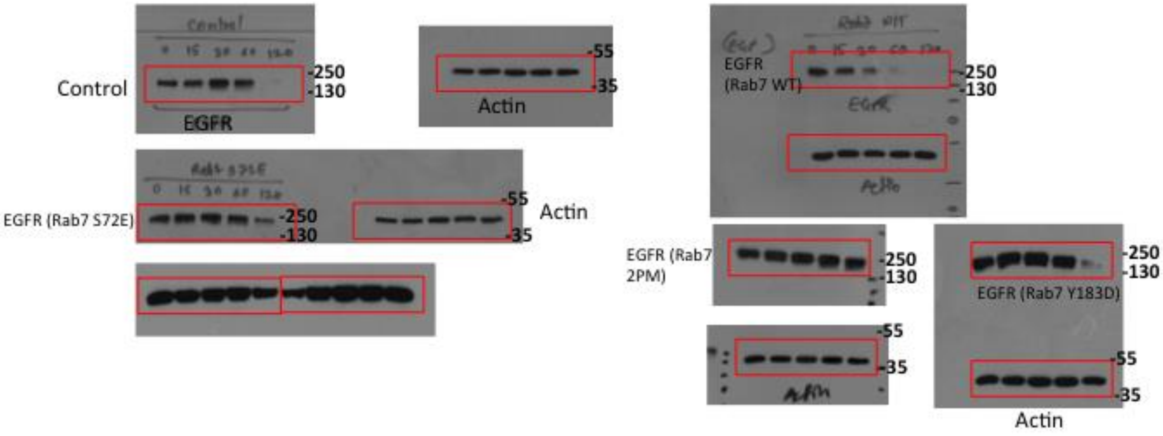

Figure 5 blots

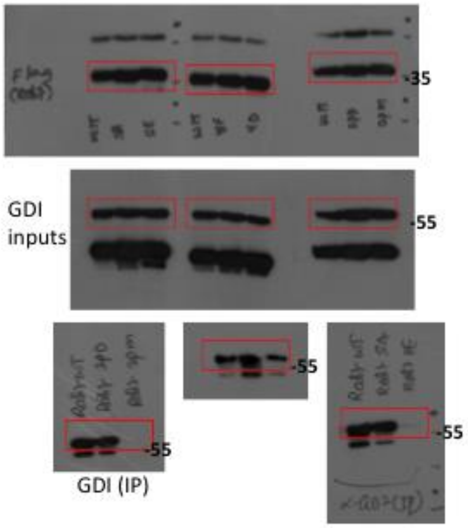

Figure 5 blots

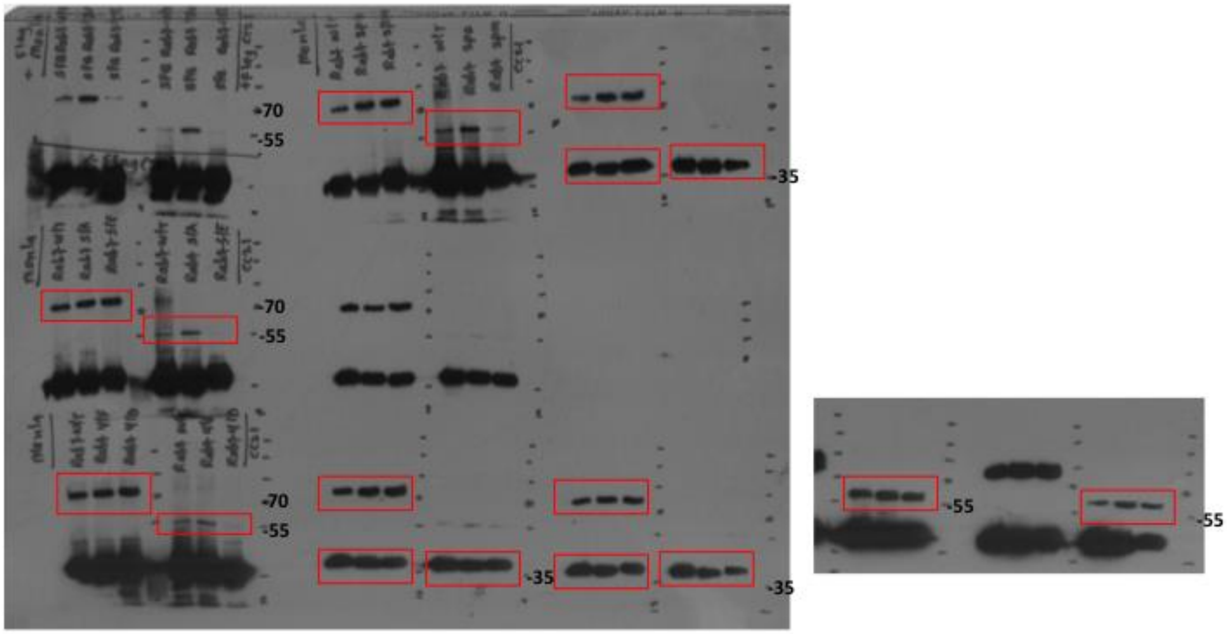

Figure 5 blots

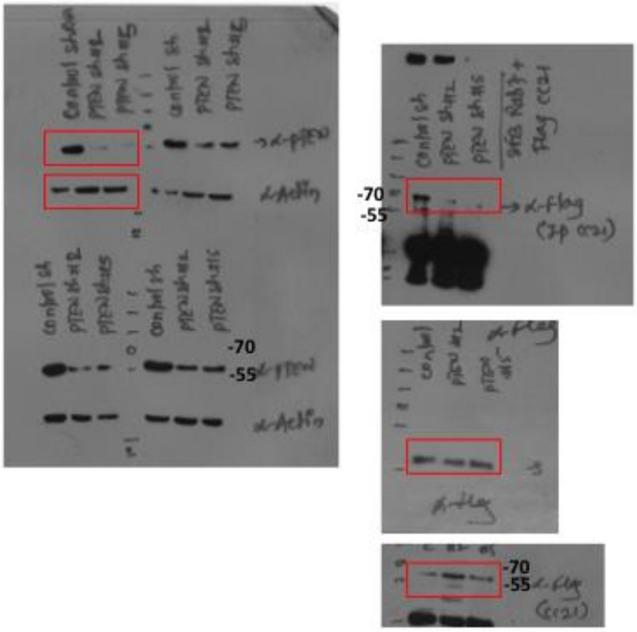

Supplementary Figure 1 blots

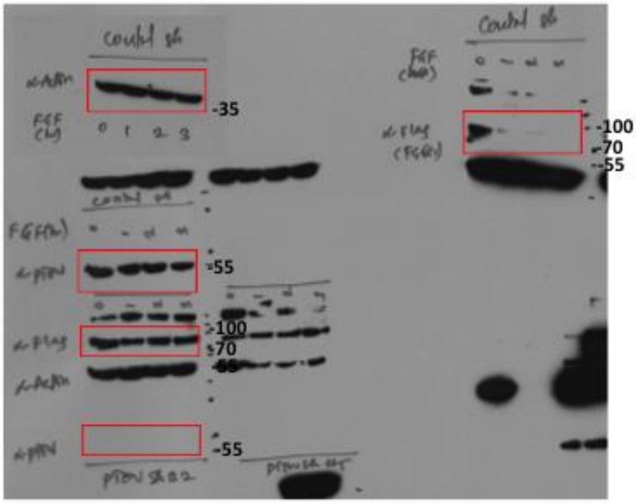

Supplementary Figure 4 blots

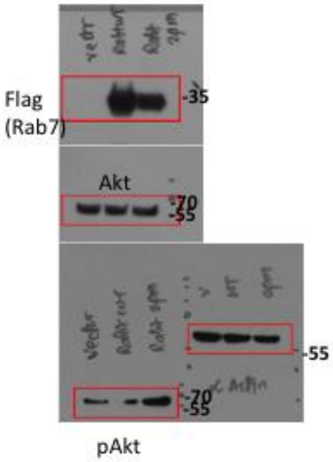

Supplementary Figure 5 blots

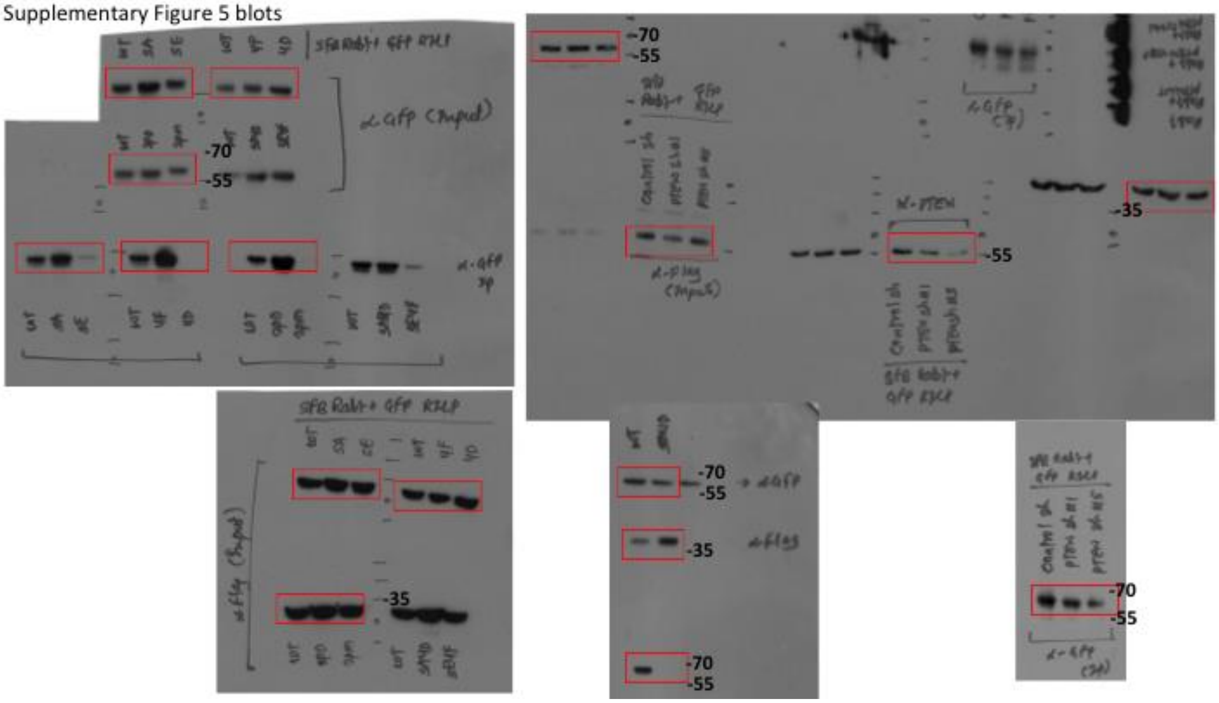

Supplementary Figure 6: Full images of the immunoblots. Red boxes show the cropped images used in the manuscript.

Table 1:

Protein	Function
PTEN	Dual specificity phosphatase
NUCL	pre-rRNA processing and ribosome assembly
PRDX1	Redox regulation
UBE1	E1 enzyme in ubiquitination process
FBX22	Substrate recognition component of SCF E3 ubiquitin ligase complex
WWP2	E3 ubiquitin ligase
Q4VJB6	Adaptor protein
PROF1	Cytoskeleton regulation
Q6IPX9	Testis specific, similar to PTEN
NDK8	Nucleotide metabolism
PAIRB	mRNA stability
PRKDC	Serine/Threonine kinase, DNA damage sensor
PPP1R10	Scaffold protein PNUTS
COF1	F-actin depolymerizing activity
MYH9	Myosin with role in cytokinesis
DYHC	Dynein motor
RAB7	Small GTPase in endo-lysosomal trafficking
GDIA	GDP/GTP exchange of Rab proteins
SYG	G-protein coupled signal transduction
GDIR	Rho GDP-dissociation inhibitor 1
MIF	Pro-inflammatory cytokine
GDIB	GDP/GTP exchange of Rab proteins
NP1L4	Nucleosome assembly protein
RUVB1	ATPase and ATP-dependent DNA helicase (3' to 5') activity
KU70	Single-stranded DNA-dependent ATP-dependent helicase
XPO1	Nuclear export
SYS	Protein trafficking
PA2G4	ERBB3-regulated signal transduction pathway
RAB1B	GTPase in vesicular trafficking

1433B	Adaptor protein
SRC8	Actin cytoskeleton organization
ANXA6	Endosome aggregation and vesicle fusion during exocytosis
LAP2A	Nuclear lamina assembly
SNX27	Sorting-Nexing member in receptor recycling
Q96CE4	Microtubule organization
MOT1	Transcription regulation
RANG	Inhibits GTP exchange on Ran
MCM4	Component of MCM helicase complex
USP9X	Deubiquitinase
WWP1	E3 ubiquitin ligase
CTDP1	Dephosphorylates CTD of RNA pol II
PPIB	Protein folding
AMOT	Tight junction maintenance
ROA2	pre-mRNA processing

Supplementary Table 1: PTEN associated proteins were identified by using tandem affinity purification followed by mass spectrometric analysis. The consolidated proteins found in PTEN interaction list from three independent purifications compared to control GFP purification were listed along with their known functions. The common contaminants such as heat shock proteins, ribosomal proteins, actin and tubulin were filtered out from the list.
